# Analysis of senescence in pituitary tumors from different lineages and the potential role of senolytic drugs as targeted therapies

**DOI:** 10.3389/fendo.2026.1836525

**Published:** 2026-07-15

**Authors:** Sergio Andonegui-Elguera, Maria J. Gonzalez-Serrano, Sophia Mercado-Medrez, Silvia Hinojosa-Alvarez, Florencia Martinez-Mendoza, Itzel Ramirez-Ramos, Leilani A. Reyna-Coahutle, Arzu Y. Sanchez-Carbajal, Stefany R. Hernandez-Bustos, Alejandro Alegria-Ortega, Jose M. Gonzalez-Mejlem, Manuel R. Garcia-Saenz, Erick Gomez-Apo, Aurea Escobar-España, Carolina Gonzalez-Torres, Javier Gaytan-Cervantes, Alam Palma-Guzman, Gerardo Guinto, Gerardo Y. Guinto-Nishimura, Blas E. Lopez-Felix, Erick U. Zepeda-Fernandez, Erick M. Estrada-Estrada, Victor Correa-Correa, Pedro A. Gonzalez-Zavala, Marco A. Asenscio-Montiel, Miguel A. Garcia-Vargas, Emmanuel Cantu-Chavez, Victor R. Chavez-Herrera, Rocio L. Arreola-Rosales, Eduardo Vadillo, Antonieta Chavez-Gonzalez, Guadalupe R. Fajardo-Orduña, Juan Jose Montesinos, Alberto Monroy-Garcia, Victor A. Cortes-Morales, Diego Mendez-Rosito, Isai Garcia-Lopez, Joshua Ramirez-Landeros, Diego A. Garcia-Fuentes, Alma Vergara-Lopez, Hugo Torres-Flores, Keiko Taniguchi-Ponciano, Daniel Marrero-Rodriguez, Moisés Mercado

**Affiliations:** 1 Unidad de Investigación Médica en Enfermedades Endocrinas, Hospital de Especialidades, Centro Médico Nacional Siglo XXI, Instituto Mexicano del Seguro Social, Ciudad de Mexico, Mexico; 2 Escuela de Ingeniería y Ciencias, Tecnológico de Monterrey, Monterrey, Mexico; 3 Programa de posgrado en Ciencias Químicobiológicas, Escuela Nacional de Ciencias Biológicas, Instituto Politécnico Nacional, Mexico City, Mexico; 4 Servicio de Endocrinología, Hospital de Especialidades, Centro Médico Nacional Siglo XXI, Instituto Mexicano del Seguro Social, Ciudad de Mexico, Mexico; 5 Área de Neuropatología, Servicio de Anatomía Patológica, Hospital General de Mexico Dr. Eduardo Liceaga, Ciudad de Mexico, Mexico; 6 Laboratorio de Secuenciación, División de Desarrollo de la Investigación, Centro Médico Nacional Siglo XXI, Instituto Mexicano del Seguro Social, Ciudad de Mexico, Mexico; 7 Laboratorio de Histología, Coordinación de Investigación en Salud, Centro Médico Nacional Siglo XXI, Instituto Mexicano del Seguro Social, Ciudad de Mexico, Mexico; 8 Centro Neurológico, Centro Médico American British Cowdray (ABC), Ciudad de Mexico, Mexico; 9 Departamento de Neurocirugia, Intituto Nacional de Neurologia y Neurocirugia, Ciudad de Mexico, Mexico; 10 Servicio de Neurocirugia, Hospital de Especialidades, Centro Médico Nacional Siglo XXI, Instituto Mexicano del Seguro Social, Ciudad de Mexico, Mexico; 11 Servicio de Patologia, Hospital de Especialidades, Centro Médico Nacional Siglo XXI, Instituto Mexicano del Seguro Social, Ciudad de Mexico, Mexico; 12 Unidad de Investigacion Medica en Enfermedades Oncologicas, Hospital de Oncologia, Centro Médico Nacional Siglo XXI, Instituto Mexicano del Seguro Social, Ciudad de Mexico, Mexico; 13 Unidad de Investigación Médica en Inmunoquimica, Hospital de Especialidades, Centro Médico Nacional Siglo XXI, Instituto Mexicano del Seguro Social, Ciudad de Mexico, Mexico; 14 Servicio de Neurocirugia, Centro Medico Nacional 20 de Noviembre, ISSSTE, Ciudad de Mexico, Mexico; 15 Servicio de Endocrinologia, Centro Medico Nacional 20 de Noviembre, ISSSTE, Ciudad de Mexico, Mexico; 16 Servicio de Anatomía Patológica, Hospital General de Mexico Dr. Eduardo Liceaga, Ciudad de Mexico, Mexico

**Keywords:** pituitary tumors, spatial transcriptomics, scRNAseq, senescence, dasatinib

## Abstract

**Introduction:**

Pituitary tumors (PT) constitute the second most frequent intracranial tumor. A subset of PT can behave aggressively despite multimodal treatment. Hallmarks such as cellular senescence, epithelial–mesenchymal transition (EMT), and stemness have been implicated in tumor progression, but their role in PT pathogenesis remains is unclear.

**Methods:**

We performed spatial transcriptomics (ST) in dopamine agonist resistant prolactin secreting PT, as well as single nucleus RNAseq (snRNAseq) in growth hormone secreting and non-functioning of gonadotropic differentiation PT to describe the senescence, proliferative and stemness landscapes. Bioinformatic analyses included clustering, senescence scoring (SenePy), cell cycle inference (ccAFv2), differentiation potential (CytoTRACE2), and EMT signature evaluation. Primary tumor cell cultures were established to validate senescence (β-galactosidase activity) and to assess the senolytic effect of dasatinib.

**Result:**

All PT included in the study were transcriptomically heterogeneous, showing between three and ten transcriptional clusters. Senescence analysis reveals two main clusters regardless of PT lineage: One with a high and the other one with low senescence score. Most spots contain terminally differentiated cells at phase G1/G0 of the cell cycle. Distinct alteration in different signaling pathways were found in clusters with low senescence score: PI3K-cascade-FGFR1 and inositol phosphate metabolism among prolactin secreting tumors, phosphatidyl inositol signaling system and serine/threonine kinase activity the in GH-secreting PT, sphingolipid signaling pathway and serine/threonine kinase alterations among non-functioning of gonadotropic differentiation PT. Dasatinib treatment of primary cell cultures of pituitary tumors of different lineages showed significant dose-dependent cell death, accompanied by caspase-3/7 activation and morphological changes consistent with apoptosis.

**Conclusion:**

Our data show that PT may contain senescent and terminally differentiated cells without any EMT evidence, and dasatinib could represent an alternative for therapy resistant PT.

## 1 Introduction

Pituitary tumors (PT) are epithelial neoplasms arising from adenohypophyseal cells and constitute the second most frequent intracranial neoplasm (1–3). Functioning PT include GH-secreting somatotrophinomas, prolactin (PRL)-secreting prolactinomas, ACTH-secreting corticotrophinomas and the rare TSH-secreting thyrotrophinomas. Non-functioning (NF) PT are usually of gonadotrope differentiation, but they seldom result in hormonal hypersecretion (1–3). The clinical, pathological, and molecular features of malignant and aggressive PT remain to be clearly defined, as pituitary carcinomas are similar to aggressive PT except for the presence of craniospinal or distant metastases (4). Although the molecular pathogenesis of aggressive PT is still largely unknown, some of the cellular changes during tumorigenesis include a senescent phenotype induced by pituitary tumor transforming gene (*PTTG*), the presence of epithelial-mesenchymal transition (EMT) related gene expression, as well as the presence of a stem cell population (5–8).

During the multistep developmental process of a neoplasm, tumor cells acquire crucial features that allow them to survive and proliferate. Such hallmarks include the ability to sustain proliferation through the action of various growth factor ligands on its cognate receptors, which trigger the activation of several signaling pathways (9). Cellular senescence, defined as a growth arrest refractory to exogenous mitogens has long been viewed as a protective mechanism against neoplasia formation (10). However, in certain contexts, senescent cells can stimulate tumor development and malignant progression (11). EMT is a cellular program essential for embryogenesis, wound healing and malignant transformation. During EMT, cell–cell and cell–extracellular matrix interactions are remodeled, which leads to the detachment of epithelial cells from each other and from the underlying basement membrane, and a new transcriptional program is activated to promote the mesenchymal fate (12). Another feature contributing to tumor development and progression is the recruitment of cancer stem cells (CSC) which have tumorigenic as well as self-renewal and differentiation abilities. Although CSC are significantly low in number, they are usually resistant to conventional chemotherapy and radiotherapy and may serve as a sanctuary of tumoral cells (13). These tumor capabilities often coexist and induction of EMT in transformed epithelial cells has been shown to culminate in endowing cells with stem-like traits (14). Activation of EMT is linked to suppression of cellular senescence since several transcription factors can both inhibit senescence and induce EMT (14).

Hallmarks such as cellular senescence, EMT, and stemness have been implicated in tumor progression across cancers, but their role in PT oncogenesis is unclear. In the present work we carried out spatial transcriptomics (ST) in therapy resistant prolactin-secreting PT as well as single nucleus RNAseq (snRNAseq) in tumors of somatotroph and gonadotrope lineage. Our aim was to describe the senescence, proliferative, EMT and stemness landscapes of therapy resistant PT. Additionally, we evaluated the *in vitro* effect of dasatinib, a senolytic tyrosine kinase inhibitor (TKI), on the induction of cellular death in primary cell cultures derived from patients with PT.

## 2 Materials and methods

### 2.1 Patients

We performed Visium ST on tumor samples from eight patients with dopamine agonist (DA)-resistant PRL-secreting PT (15). One patient presented to the otorhinolaryngology department with a sphenoid sinus mass, which was biopsied and confirmed as a PRL-secreting lesion documented by MRI; she was treated with cabergoline. The remaining seven patients had been subjected to transsphenoidal surgery because of lack of response to DA treatment. Response to cabergoline treatment was defined as follows: complete response, normalization of PRL levels and > 50% reduction of tumor mass; partial or stable response, > 50% reduction in PRL levels and any reduction in tumor mass; and null response, less than 50% reduction in PRL levels and no tumor mass reduction (15–17).

snRNAseq was carried out in six patients with PT, two GH-secreting and four NF PT of gonadotropic differentiation. All patients had undergone transsphenoidal surgery. A tumor was categorized as aggressive when after primary pituitary surgery it required additional treatments such as radiotherapy, chemotherapy, or repeated surgeries due to rapid tumor growth (18–20).

All tumors included in the study were sporadic and were collected from patients who had been diagnosed, treated and followed at the Endocrinology Service and the Neurosurgical department of Hospital de Especialidades, Centro Médico Nacional Siglo XXI of the Instituto Mexicano del Seguro Social. All participating patients were recruited with signed informed consent and ethical approval from the Comisión Nacional de Ética e Investigación Científica of the Instituto Mexicano del Seguro Social in accordance with the Helsinki declaration (15).

### 2.2 Immunophenotyping of PT: immunohistochemistry for hormones and transcription factors

Immunohistochemistry (IHC) was performed as previously described (21). Briefly, paraffin-embedded, formalin-fixed tissue blocks were obtained and 3 μm sections were stained with hematoxylin-eosin (H&E) and reviewed by a neuropathologist. Tumors were represented with a 2-fold redundancy. Sections were cut and placed onto coated slides. Immunostaining was performed by means of the HiDef detection HRP polymer system (Cell Marque, CA, USA), using specific antibodies against hormones and transcription factors with a 1:100 dilution: TSH (CM412B, BioCare Medical, CA, USA), GH (A0570, Dako, CA, USA), PRL (A0569, Dako, CA, USA), FSH (M3504, Dako, CA, USA), LH (M3502, Dako, CA, USA), ACTH (M3501, Dako, CA, USA), TBX19 (AB243028, Abcam, CA, UK), POU1F1 (NBP1-92273, Novus Biologicals, CO, USA), NR5A1 (SC393592 Santa Cruz Biotechnology TX, USA) and MKI67 (NB500-170, Novus Biologicals, CO, USA). All IHC reactions were performed overnight at 4°C in a humidity chamber. Interpretation of IHC was carried out by two independent observers.

### 2.3 Visium spatial transcriptomics

ST was carried out in representative areas of H&E slides from each tumor, previously reviewed by a neuropathologist (15). Visium Spatial Gene Expression for FFPE V1 (1000338, 10X Genomics) instructions were followed as specified by manufacturers. The FFPE tissue blocks were cut 5 μm-thick as recommended. Slices were placed onto Visium Spatial Gene Expression Slide, and the tissue was deparaffinized at 60°C for 2 h and xylene baths followed by decreasing concentrations of ethanol and finally molecular grade water. Once the tissue was deparaffinized and re-hydrated it was stained with H&E. The tissue images were digitalized with the Aperio CS2 by Leica. Decrosslinking was performed with 0.1N HCl as recommended. Libraries were constructed with the Visium Spatial Gene Expression Reagent Kit as follows, FFPE probes were hybridized overnight and subsequently were ligated, the RNA was digested, and the probes released for extension and eluted for amplification and index ligation and finally library cleanup. The libraries were pair-end sequenced in a NextSeq2000 (15).

### 2.4 Visium ST bioinformatic analysis

The bioinformatic analysis was conducted using Ubuntu 22.04.5 LTS, based on Linux. Statistical analyses and figure constructions were carried out using R version 4.4.1, unless otherwise specified as in the following sections (15). Data preprocessing was performed using Space Ranger 2.1.1 with GRCh38 as the reference genome. For further analysis, the Seurat package was used, whereby data was normalized with the “SCTransform” function using default parameters on the “Spatial” assay, followed by dimensionality reduction and clustering. Variable features were calculated using the “FindAllMarkers” function and “FindSpatiallyVariableFeatures” for spatial variogram variable features (15).

SCTransform-normalized data were used to calculate senescence scores with the SenePy package version 1.0.1 trough Anaconda environment and employing the “Universal” signature. Based on these scores, previously identified clusters were classified into High- and Low-senescence groups. Differentially expressed genes between these groups were identified using the FindMarkers() function from Seurat package version 5.3.0. Functional enrichment analyses were then performed with the enricher() and GSEA() functions from the ClusterProfiler package version 4.14.6, considering only genes with an adjusted p-value < 0.05 and an absolute logFC > 0.5.

SCTransform-normalized data were used to classify spatial transcriptomic spots into cell cycle states. The ccAFv2 algorithm was applied to classify cells according to the following five phases: G0/G1, S, G2/M, M/Early G1, and Unknown, using the PredictCellCycle() function from the ccAFv2 R package version 0.0.0.9 installed via GitHub, with the parameter spatial = TRUE and all other parameters set to default. Cellular developmental potential was inferred using the CytoTRACE algorithm, which estimates differentiation states ranging from totipotent to pluripotent. The cytotrace2 function from the CytoTRACE2 R package version 1.1.0 was used with default parameters.

Gene signature for epithelial-mesenchymal transition (Hallmark_EMT) was obtained from MSigDB. Genes expressed in our dataset were retained and module scores were computed with Seurat’s AddModuleScore function. Scores were centered to allow direct comparison across programs, and UMAP projections were generated to visualize the distribution across cell populations.

### 2.5 Nuclei isolation from pituitaries

Each pituitary tumor tissue was processed as an individual single nuclei multiome reaction (22). The sample was pulverized, and a fraction of the resulting tissue powder was used for nuclei isolation. The remaining material was returned to -80 °C for storage. All subsequent procedures were performed on ice. Firstly, an RNAse inhibitor (NEB, Ipswich, MA, USA; cat# M0314L) was added to the homogenization buffer (0.32 M sucrose, 1mM EDTA, 10mM Tris-HCl pH 7.4, 5 mM CaCl_2_, 3mM Mg (Ac)_2_, 0.1% IGEPAL CA-630), and OptiPrep solutions at 50%, 35%, and 30% were prepared from a 60% stock (StemCell, Canada; cat# 07820). Each sample was then homogenized in a 1mL Dounce glass homogenizer (VWR, Radnor, PA, USA; cat# 71000-514) and passed through a 40 mm cell strainer. We then added an equal volume of 50% OptiPrep to the filtrate which was centrifuged in an SW41 rotor (17, 792 xg; 4 °C; 25 min). Nuclei were recovered from the interphase, washed, resuspended in 1X nuclei dilution buffer, and counted with a Cellometer.

### 2.6 Library preparation and single nucleus multiome assay

The sn multiome assay was carried out according to the Chromium Single Cell Multiome ATAC and Gene Expression Reagent Kits V1 User Guide (10x Genomics, Pleasanton, CA) (22). Nuclei were counted using propidium iodide fluorescence with a Nexcelom Cellometer (Nexcelom Bioscience, Lawrence, MA, USA). Transposition was performed in 10 µl reactions at 37 °C for 60 minutes, using 2 000 – 20–000 nuclei, prior to loading onto the Chromium Chip J (10x Genomics, Pleasanton, CA; PN-2000264) for GEM generation and barcoding. After GEM cleanup, libraries were pre-amplified by PCR and the material was divided into three fractions: one for snRNAseq library construction, one for snATACseq library construction, and a remaining fraction was stored at -20 °C. Both snRNA and snATAC libraries were indexed for multiplexing using Chromium i7 Sample Index N, Set A (10x Genomics, PN-3000262) and Chromium i7 Sample Index TT, Set A (10x Genomics, PN-3000431), respectively.

### 2.7 Quality control and libraries sequencing

Libraries were quantified using a Qubit 3 fluorometer (Invitrogen, CA, USA), and quality was evaluated with a Bioanalyzer (Agilent Technologies, CA, USA) (17). Libraries were then pooled at equivalent molar concentrations, and read distribution was adjusted after an initial sequencing run on a Miseq (Illumina, CA, USA). Final sequencing was performed on a Novaseq 6000 (Illumina) at the New York Genome Center (NYGC) and the New York University Technology Center, following Illumina and 10X Genomics guidelines (22).

### 2.8 Single nucleus RNA sequencing bioinformatic analysis

Data was processed using Cell Ranger ARC version 1.0.0 (10X Genomics) with default parameters. Cellular barcodes were assigned, and sequencing reads were aligned to the human reference genome (hg38), following 10X Genomics guidelines. Filtered feature-barcode gene expression (GEX) matrices per sample were imported to R v4.4.0 (23) using the Seurat package v5.1.0 (24) to create a Seurat object. Cells not meeting the following quality control thresholds were excluded: GEX counts between 1–000 and 25 000; mitochondrial content below 10%. Additionally, violin plots were generated to visualize mitochondrial read percentage, to ensure de absence of cells with high mitochondrial DNA content. Normalization of the GEX matrix was performed using SCTransform (25), followed by dimensionality reduction with PCA.

For clustering, the nearest neighbors were computed using the FindNeighbors function based on a maximum likelihood approach. An elbow plot was then generated to determine the optimal number of dimensions for PCA. The first 10 dimensions were used, and clustering was performed with the Leiden algorithm at a resolution of 0.5. Visualization was carried out using UMAP (Uniform Manifold Approximation and Projection). Cell types were manually annotated.

Senescence scores were computed with the SenePy Python package (26). Human senescence hubs were loaded and merged into a single universal signature. This standardized reference enabled consistent scoring and comparison of senescence signatures across samples in downstream analyses. After obtaining senescence scores per sample, cells were classified into high and low senescence, using a sample-specific threshold, according to the median senescence value of each sample.

Cells were classified into high and low senescence groups, and differential gene expression analysis was performed between the two categories using the Wilcoxon Rank Sum test in Seurat’s FindMarkers function. Genes were considered significantly differentially expressed when they met both criteria: Benjamini-Hochberg adjusted p value < 0.05 and an absolute log2 fold change greater than 0.2. Functional enrichment was then performed with CusterProfiler v4.14.6 (27) evaluating Gene Ontology categories (biological processes, molecular functions, and cellular components), as well as KEGG and Reactome (28) pathways.

To infer cellular differentiation potency, the cytotrace2 function from the CytoTRACE2 R package v1.1.0 (29) was applied using default parameters. Cells were assigned to categorical potency states: Totipotent, pluripotent, oligopotent, multipotent, unipotent. Cell cycle classification was performed using the PredictCellCycle function from the ccAFv2 package v0.0.0.9 (30), assigning cells to G1, Late G1, S, S/G2, G2/M, or M/Early G1 phases.

Gene signature for epithelial-mesenchymal transition (Hallmark_EMT) was obtained from MSigDB. Genes expressed in our dataset were retained and module scores were computed with Seurat’s AddModuleScore function. Scores were centered to allow direct comparison across programs, and UMAP projections were generated to visualize the distribution across cell populations.

### 2.9 Primary tumor cell cultures

PT tissue samples were surgically obtained and immediately enzymatically dissociated to establish primary tumor cell cultures (8 non-functioning, 1 corticotroph, 1 PRL- and 1 GH-secreting tumor). Samples were washed with HBSS and incubated at the thermoshaker for 60 minutes at 37°C and 300 rpm with 0.5 mg/mL of collagenase II and IV each (Thermo Scientific). Cells were passed through a 30 μm mesh and the pellet was obtained after centrifugation followed by red cell lysis buffer washes. Live and viable cells were enriched with the EasySep Dead Cell Removal (Annexin V) Kit (Stem Cell Technologies) to eliminate dying and dead cells. Viable primary tumor cells derived from patients were cultured in a 1:1 ratio of DMEM-F12 and DMEM-High glucose with 15% fetal bovine serum with 1% penicillin/streptomycin at 37 °C in 5% CO2.

### 2.10 CCK8 viability assay

To assure a viable cell culture before any experiment, cells were seeded 50, 000 per well in a 96-well plate and exposed to the CCK8 reagent (Abcam, UK) immediately after dissociation. They were then incubated for 24, 48, and 72 hours. Following each incubation period, absorbance was measured at 460 nm using a plate reader. Absorbance values were analyzed comparing them to a well of cell culture medium along with CCK8 reagent, determining tetrazolium salt metabolization by the cellular mitochondrial dehydrogenases.

### 2.11 Dasatinib challenge and apoptosis assay

Primary tumor cells were seeded at 50, 000 per well in a 96-well plate and incubated for 24 hours. After incubation, cells were treated with increasing concentrations of dasatinib (2.5 and 5 μM) and incubated for 24 or 48 hours.

For the cell viability and cell death identification we used the LIVE/DEAD viability/cytotoxicity assay kit (green/deep red) (Invitrogen), cells were stained with calcein and SYTOX after 24 hours of dasatinib administration, to determine cell viability and death, respectively. In order to confirm apoptosis by caspase activation on the primary cultures, CellEvent Caspase-3/7 green ready probes reagents (Invitrogen) were applied to the cultures along with dasatinib and then they were incubated for 24 hours.

We also use Att-20, a corticotroph-derived tumor murine cell line to corroborate the observed cell death induction in GH3 cell line previously described (21) by measuring caspase 3/7 activation. Briefly, 50, 000 Att-20 cells per well were seeded in a 96-well plate. After 24 hours, a caspase 3/7 detection reagent (CellEvent Caspase-3/7 green ready probes reagents, Invitrogen) was added along with 2.5 and 5 µM dasatinib. Then, cells were incubated for 24 hours and assessed using an EVOS M3000 fluorescence microscope at 10× magnification with a GFP filter.

### 2.12 Statistical analysis

Shapiro-Wilk test (n=8) was used to assess the distribution of variables. The data did not follow a normal distribution; therefore, non-parametric statistical tests were applied, and vafriables were presented as medians with interquartile ranges (IQR). Association between the high and low senescence score, Ki-67 staining and clinical variables were evaluated using Spearman’s rank correlation coefficient, comparisons between independent groups were performed using the Mann-Whitney U test. Statistical analyses were conducted using IBM SPSS Statistics v.27, considering p < 0.05 as statistically significant.

## 3 Results

### 3.1 Clinical and demographic characteristics of the patients

**Supplementary Table 1** summarizes the demographic, clinical, and biochemical characteristics of eight patients with therapy-resistant prolactinomas that had been subjected to ST (15). snRNAseq was carried out in six PT, two somatotrophinomas and four nonfunctioning gonadotrophinomas and their clinical, biochemical and imaging characteristics are summarized in **Supplementary Table 2**. The median age was 46 years (IQR 36–58); one was a woman and five were men. At diagnosis, all presented with visual field defects, and two presented with pituitary apoplexy. The median tumor size was 34.5 mm (range: 26.2–38.3), and 83% of patients showed cavernous sinus invasion. According to our predefined criteria, two patients presented with aggressive disease: a patient with a GH-secreting PT who required fractionated radiotherapy and octreotide treatment and a patient with a nonfunctioining PT, who required fractionated radiotherapy as an additional treatment after surgery.

#### 3.1.1 Senescence landscape of therapy-resistant prolactinomas

**Figure 1:**
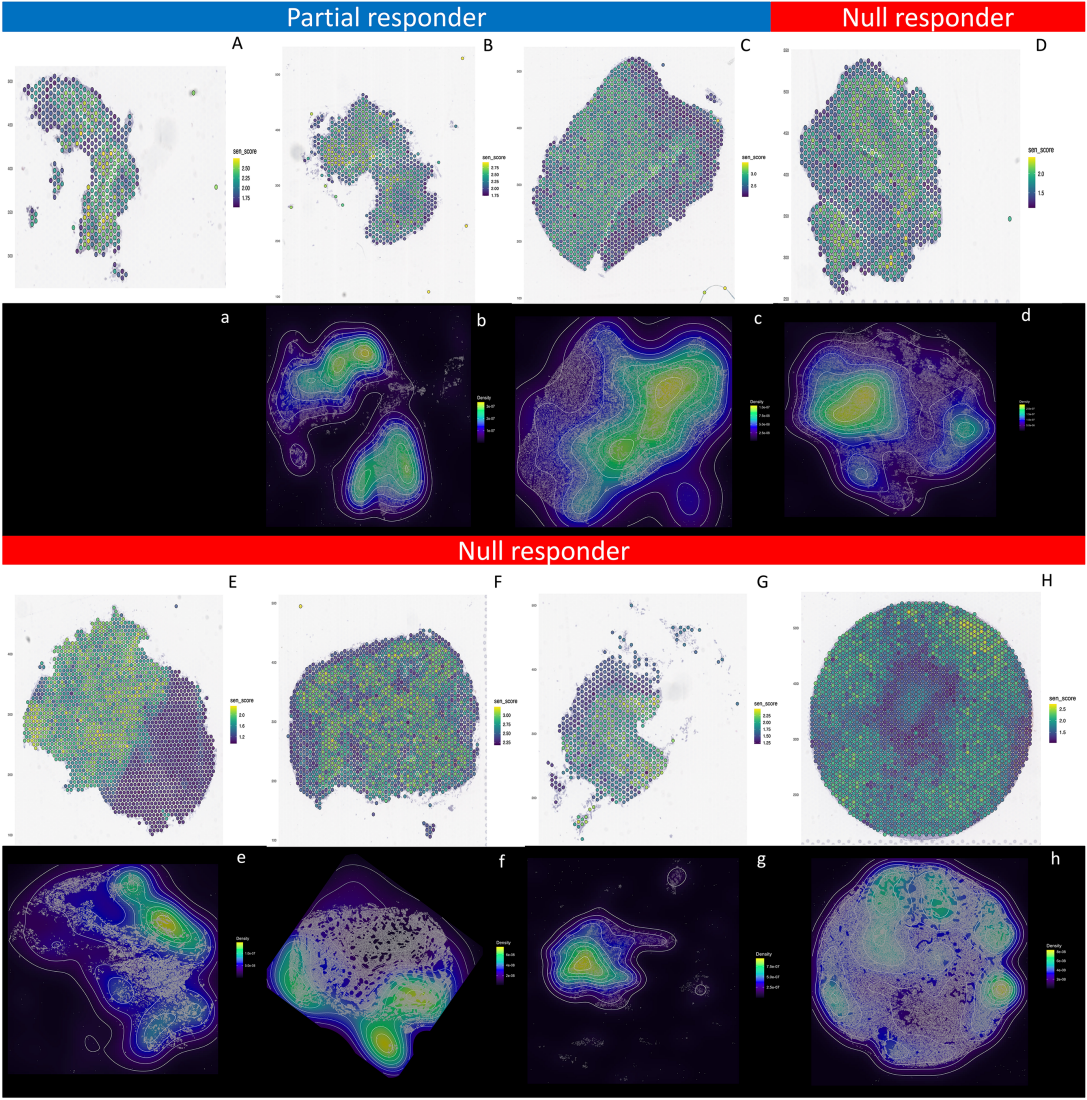
**(A–C)** show the partial responder PT with the senescence scores. Dark blue color depicts lower senescence score, whereas green and yellow colors depict higher senescence scores calculated with Senepy. **(b, c)** show the intensity map of Ki67 immunohistochemistry staining, the purple and dark blue color depict less density of Ki67 positive cells, whereas the light blue, green and yellow colors depict higher density of Ki67 positive cells. The **(D–H)** show the null responder PT with the senescence scores, whereas **(d–h)** panels show the intensity map of Ki67 immunohistochemistry staining in null response PT. Some high Ki67 staining density areas correlate with lower senescence scores in several tissues.

**Figure 2:**
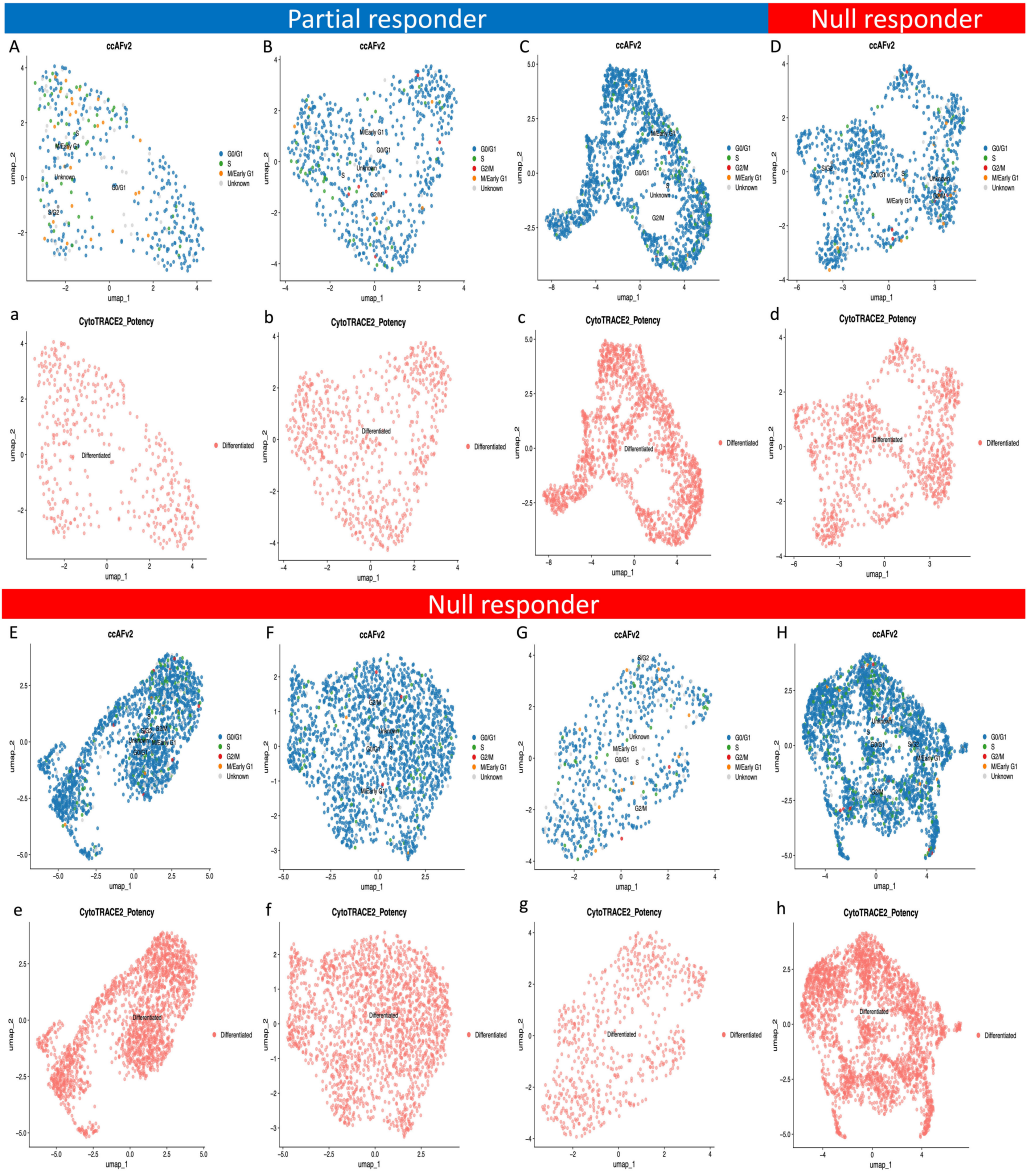
**(A–C)** show the partial responder PT are stalled at G1/G0 cell cycle phase. PT from partial responder patients showing cells stalled at G1/G0 cell cycle phase **(a–c)** show the partial responder PT terminally differentiated profiles. **(D–H)** show the null responder PT spots at G1/G0 cell cycle phase, whereas **(d–h)** panels, again, show terminally differentiated profiles.

We have previously reported the depth and spot sequencing results of eight patients with lactotroph PT (15). We sequenced a total of 11054 spots with 107473 mean reads per spot with more than 95% of reads mapped correctly to the reference, showing a high intratumoral cellular heterogeneity (15). Each individual tumor was found to have 3 to 10 cell clusters comprising the tumor mass. One of the three patients categorized as having a partial response to cabergoline harbored a tumor with only three cell clusters, whereas one of the five null responders had tumors consisting of up to 10 cell clusters (**Figures 1A–H**) (15). We first performed SenePy analysis to evaluate the senescence landscape in PT. Despite the heterogeneity observed in transcriptomic clusters, SenePy analysis revealed that all tumors harbored a low and a high senescence cluster. The vast majority of the spots in all tumor samples showed a high senescence score (**Figures 1A–H**). In the majority of samples tumor areas containing low senescence clusters had a higher Ki67 proliferative index by IHC (**Figures 1b–h**). Also, most of the spots containing stem cells were located within the highly senescent clusters (**Supplementary Figure 1**). Most of the spots in all tumors showed that they were stationed in the G1/G0 phase of the cell cycle (**Figures 2A–H**).

Larger tumors showed lower senescence scores (p=0.047) while tumors from patients requiring higher DA doses showed higher senescence scores (p=0.049), yet no statistically significant differences between senescence scores among partially and null responders (p=0.655 for high senescence, p=0.653 for low senescence). No other correlations were found between senescence scores and clinical variables such as age (p=0.693), prolactin levels at diagnosis (p=0.823), prolactin levels after DA treatment (p=0.651).

Ki67 proliferative index did not correlate with age (p=0.772), baseline prolactin levels (p=0.620), prolactin levels after DA treatment (p=0.441) and maximum cabergoline dose (p=0.414). Tumors partially responsive and completely unresponsive patients have similar Ki67 indexes (p=0.786).

Classical senescence markers such as β-galactosidase (GLB1) and p27 (CDKN1B) were all expressed in both, high and low senescence clusters, with a slightly higher expression in clusters with a high senescence profile (**Supplementary Figure 2**). Other genes related to the senescence-associated secretory phenotype (SASP) such as IL-6, CXCL8, IFNɣ, MMP1, IL-1A or TGFβ were not significantly expressed in neither the low nor the high senescence clusters, whereas MIF and CTSB were present in all cell clusters (**Supplementary Figure 2**). The correlation between the senescence score and the differentiation state of the pituitary cells was evaluated by CytoTRACE2 analysis. All clusters were comprised by terminally differentiated cells (**Figures 2a–h**), which coincides with our spot deconvolution analysis that revealed a very low number of stem cells (expressing SOX2, LGR4 and RBPMS) and a high number of tumor cells (expressing PRL and POU1F1), with a relatively low number of endothelial cells (expressing VWF and CD34) and immune response cells, particularly macrophages (expressing PTPRC and CD68) (**Supplementary Figure 1**).

**Figure 3:**
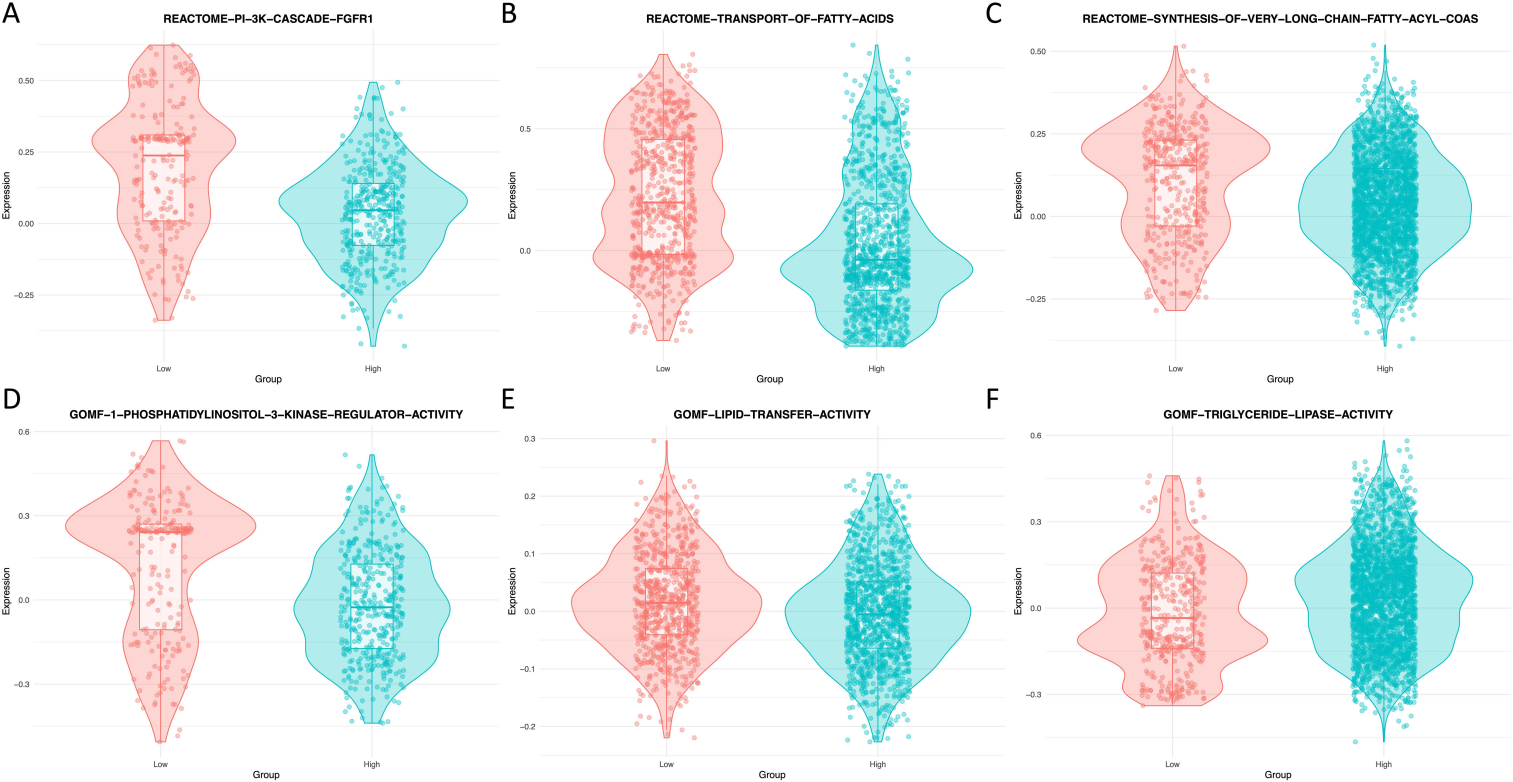
**(A–C)** show reactome enriched pathways PI3K-cascade-FGFR1, transport of fatty acids, and synthesis of very long chain fatty acyl coas, Reactome enriched pathway of low senescence tumors shoing alterations in PI3K cascade-FGFR1, transport of fatty acids, synthesis of very long chain fatty acyl CoA **(D–F)** show gene ontology molecular function altered pathways PI3K regulator activity, lipid transfer activity and triglyceride lipase activity, alterations observed in low senescence score cells. Gene ontology molecular function of tumors with a low senescence score showung alterations in lipid transfer activity, PI3K regulator avtivity and triglyceride lipase activity

Neither the high, nor the low senescence score spots significantly expressed genes related to EMT such as TWIST2 and SNAI1 (**Supplementary Figure 3**). A more in-depth analysis by using Hallmark_EMT geneset confirmed the absence of EMT profiles in the aggressive and therapy resistant lactotroph PT (**Supplementary Figure 4**). Interestingly, some of the tumors showed a high Ki67 whereas others were negative or had a low positive staining regardless of their response to therapy and the stage of cell cycle. The enrichment analysis did not show a well-defined difference between pathways in the high senescence compared to the low senescence groups. Gene ontology and Reactome databases showed that the altered events were related to lipid metabolism in the low senescence groups. Such metabolic processes included the PI3K-cascade-FGFR1, inositol phosphate metabolism, transport of fatty acids, beta oxidation of butanoyl−coenzyme A to acetyl−coenzyme A, and synthesis of very long chain fatty acyl coas and lipid binding (**Figures 3A–C**), consistent with our previous results (15, 21). The gene ontology molecular function database confirmed that the main alterations are related to PI3K regulator activity, as well as lipid transfer activity and triglyceride lipase activity (**Figures 3D–F**).

### 3.2 Somatotroph and gonadotroph PT snRNAseq senescence profile

**Figure 4:**
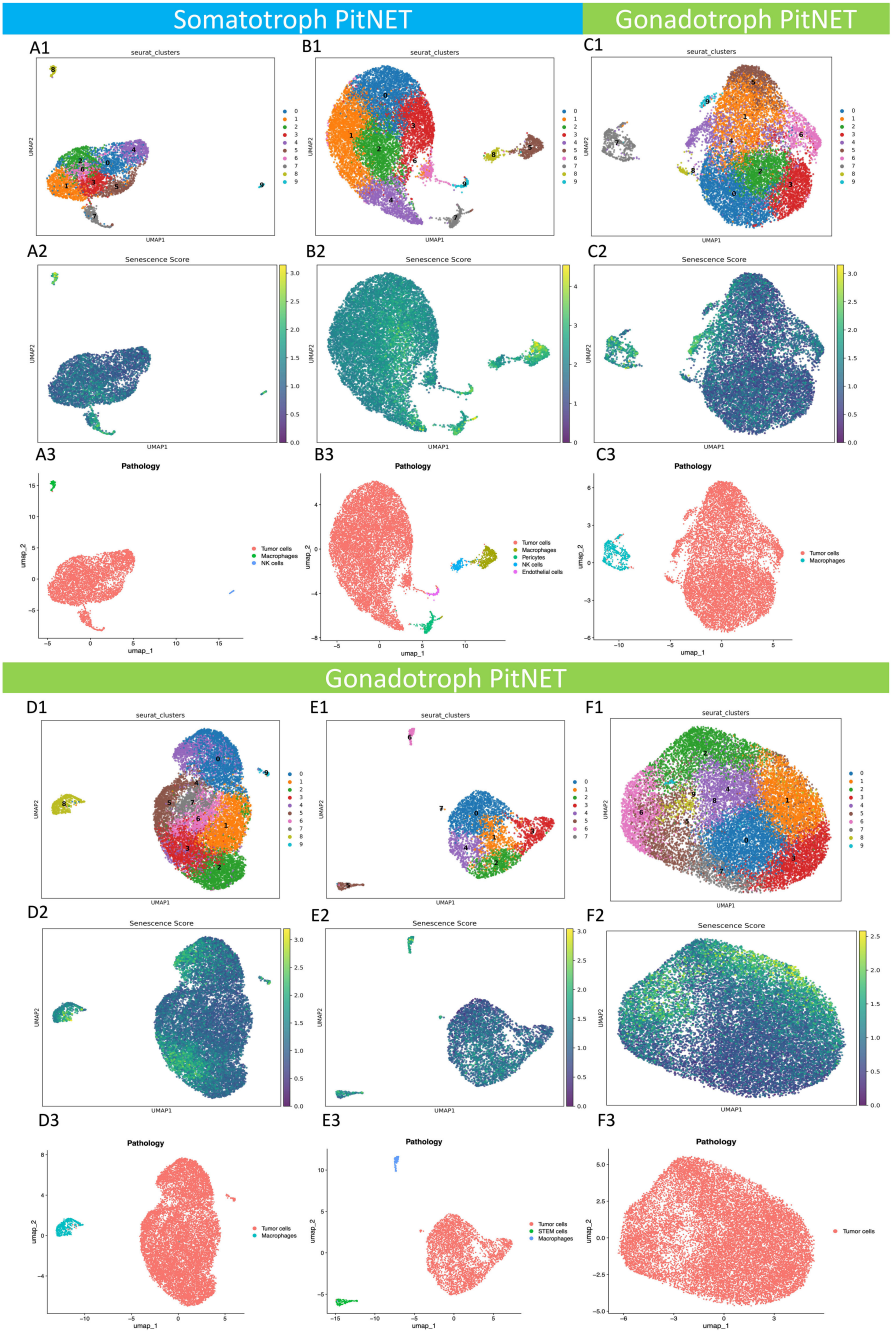
**(A1–B1)** show the transcriptomic heterogeneity present in GH-secreting PT with 10 clusters each tumor. **(C1–F1)** show the NF-gonadotroph PT with eight to ten transcriptional clusters. The **(A2–B2)** show the GH-secreting PT senescence landscape with marked difference between samples. **(C2–F2)** show the NF-gonadotroph PT with different levels of senescence between samples. All samples depict two cell clusters, one with a high and a second with a low senescence score. **(A3–F3)** depict the cell identity of the cells comprising the tumor mass.

We performed snRNAseq on somatotroph and gonadotroph PT to assess if senescence was also present in other tumor lineages. For this experiment, we sequenced a total of 62, 460 cells. The two somatotroph PT and all but one gonadotroph tumors showed 10 transcriptional clusters. The remaining gonadotrophe PT consisted of 8 transcriptional clusters (**Figures 4A1–F1**).

**Figure 5:**
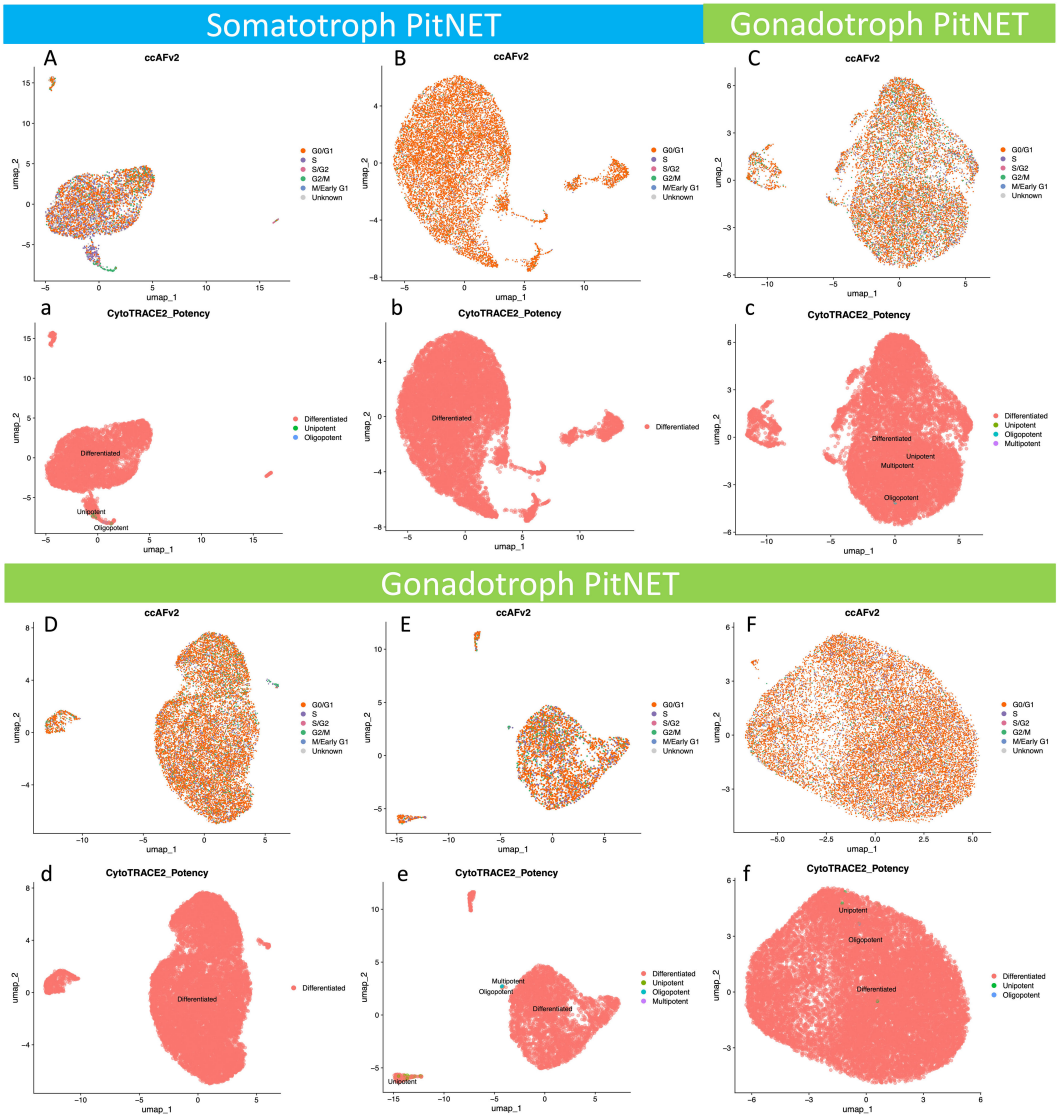
**(A, B)** show GH-secreting PT G1/G0 cell cycle phase predominance, **(A)** corresponds to the aggressive somatotroph PT and contains more cells at S phase of the cell cycle. **(a, b)** show terminally differentiated cells in GH-secreting PT. **(C–F)** show the NF-gonadotroph PT stationed at G1/G0 cell cycle phase and **(c–f)** show terminally differentiated tumor cells.

snRNAseq analysis showed that, despite marked intratumoral transcriptional heterogeneity, cells consistently segregated into two principal clusters defined by their senescence status: a high- and a low-senescence group. The GH-secreting and the NF-gonadotroph PT differ in their senescence profiles when compared to the lactotroph PT, showing a higher heterogeneity across samples in both tumors involving only tumoral cells. In NF-gonadotroph PT, two non-aggressive and the aggressive tumor samples were predominantly composed of cells with low senescence scores, representing 80.7%, 73.7%, and 70.1% of cells, respectively. In contrast, one non-aggressive sample exhibited a higher proportion of cells with high senescence scores (56.9% of cells). The senescence profiles between the two GH-secreting tumors were substantially different: the aggressive tumor contained a higher number of cells with low senescence scores (65.8% of cells), whereas the non-aggressive tumor was almost entirely composed of cells with high senescence scores (94.2% of cells) (**Figures 4A2–F2**). Expression of GLB1 and CDKN1B was detected across both clusters. In contrast, canonical SASP genes—including IL6, CXCL8, IFNγ, MMP1, IL1A, and TGFβ—were not expressed. MIF, however, was uniformly detected across all clusters, independently of SASP status, in both somatotroph and gonadotroph PT. Cell cycle analysis indicated that most tumor cells were stationed in the G0/G1 phase (**Figures 5A–F**).

CytoTRACE2 analysis further supported a predominantly terminally differentiated cellular landscape, with only a minor fraction of oligopotent and multipotent cells (**Figures 5a–f**). This finding aligns with cell-type annotation, which demonstrated that tumor mass is largely composed of differentiated tumor cells—expressing GH and POU1F1 in somatotroph tumors, and FSH/LH and NR5A1 in gonadotroph tumors. Stem-like cells (positive for SOX2, LGR4, RBPMS) were scarce and confined to a single tumor. Non-tumoral populations, including endothelial cells (VWF, CD34), pericytes (PDGFRB, CSPG4, NES), and immune cells—particularly macrophages (PTPRC, CD68)—were present at relatively low abundance (**Figures 4A3–F3**). EMT-associated transcription factors TWIST2 and SNAI1 were detected at low levels in a very limited number of cells. Consistently, Hallmark_EMT gene set analysis revealed no significant enrichment across senescence clusters or tumor types (**Supplementary Figure 5**).

**Figure 6:**
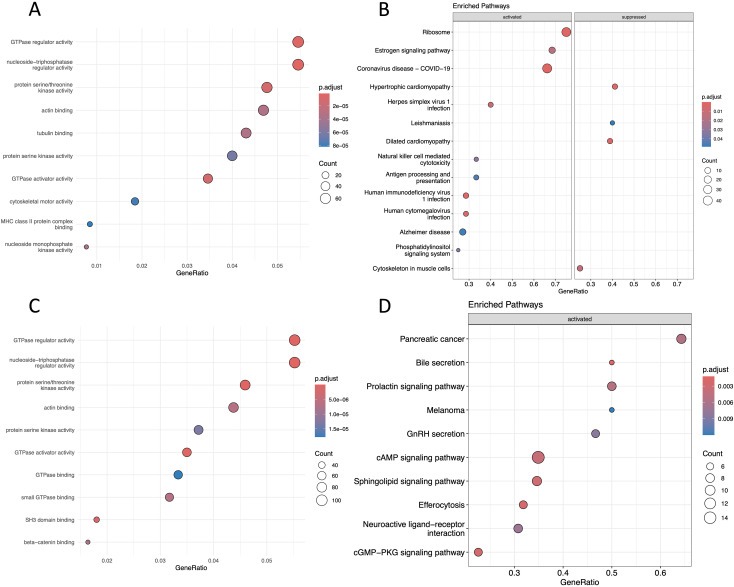
**(A, B)** Altered pathway in low senescence score clusters from the snRNAseq data from somatotroph PT showed alterations in phosphatidyl inositol signaling system and serine/threonine kinase activity among other events, and **(C, D)** NF-gonadotroph PT show altered pathways related to sphingolipid signaling pathway and serine/threonine kinase activity among other events.

The differential gene expression between the low and the high senescence score groups and subsequent enrichment from the snRNAseq data from somatotroph PT showed alterations in phosphatidyl inositol signaling system and serine/threonine kinase activity (**Figures 6A, B**) among other events. Gonadotroph PT showed that most of the altered pathways are related to sphingolipid signaling pathway and serine/threonine kinase activity (**Figures 6C, D**).

GH-secreting tumors exhibited higher senescence levels compared to NF-gonadotroph tumors; however, this difference did not reach statistical significance (p=0.165). In addition, differences in senescence levels between aggressive and non-aggressive tumors were not statistically significant (p=1.000). Similarly, no statistically significant differences in high or low senescence levels were observed between aggressive and non-aggressive tumors (p = 1.000 both). Tumors with cavernous sinus invasion showed higher proportions of high senescence scores without reaching statistical significance (p=0.143). Moreover, maximum tumor diameter tended to be negatively correlated with high-senescence levels, again, without reaching statistical significance (p=0.397). There was a tendency for a negative correlation between age at diagnosis and positive Ki-67 (p = 0.468), suggesting that tumors in younger patients may exhibit slightly higher proliferative activity.

### 3.3 Primary tumor cell culture, senescence confirmation and repurposing dasatinib as a senolytic targeting drug in PT

**Figure 7:**
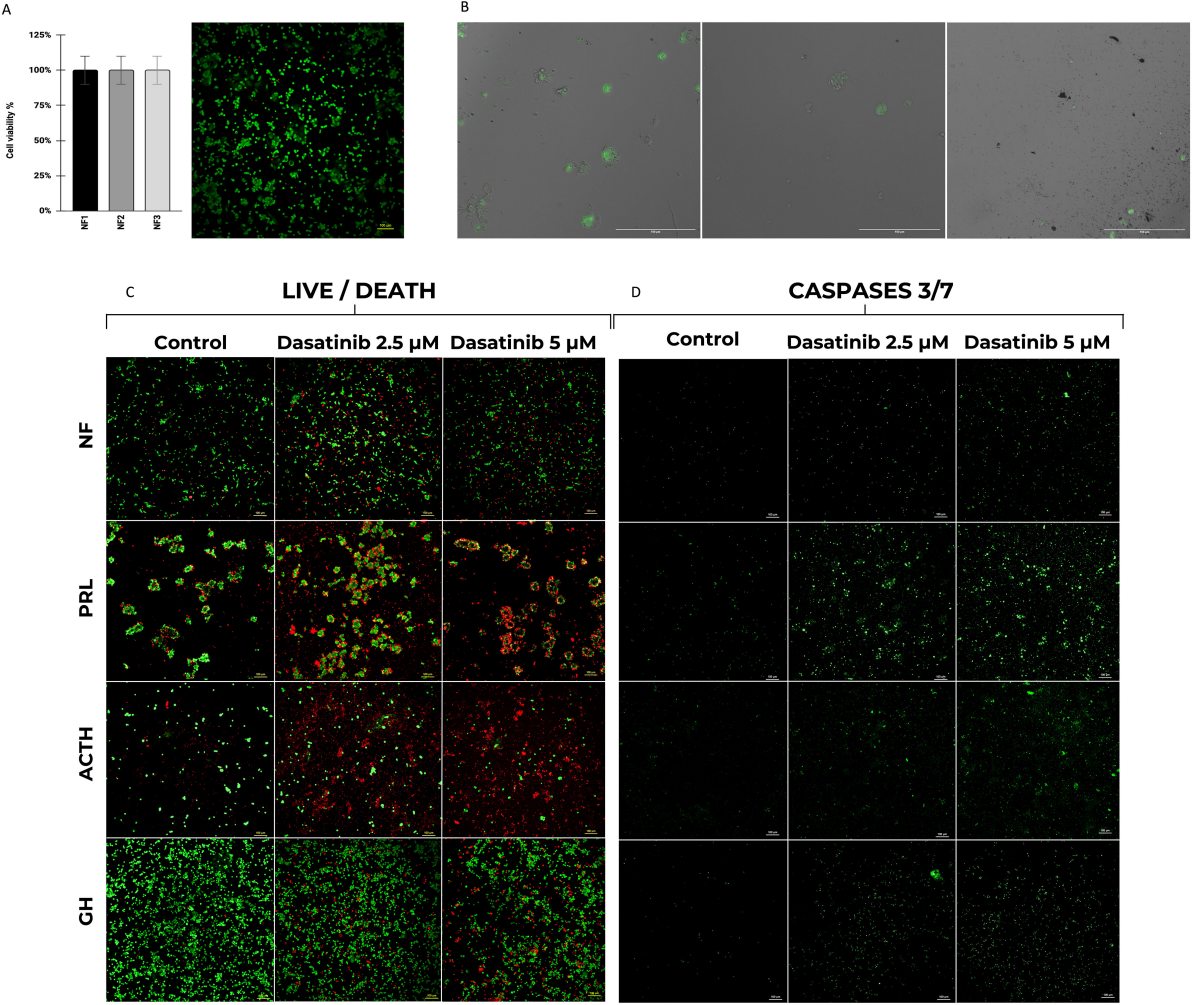
**(A)** shows high viability in pituitary tumor primary cell culture measured by WST and calcein staining, represented by bar graph and green fluorescence image respectively. **(B)** shows the positive green fluorescence cells corresponding to metabolic activation of β-galactosidase an indirect marker of senescence, and the posterior elimination of positive tumor cells by dasatinib. **(C)** show live/dead cell staining results, it is observed shoing a dose-dependent the increment of dead cells (red staining) in a dose-dependent effect induced by dasatinib exposure and inversely the decrement of live viable cells (green staining) in a dose dependent manner, and **(D)** show the increased activation of caspase 3/7, again in dose dependent manner to dasatinib exposure, confirming cell death shows a dose-dependent increased activation of casoase 3/7 induced by dasatinib exposure. **(C, D)** results correspond to primary cell culture derived from NF-gonadotroph, PRL-, GH- and ACTH-secreting pituitary tumors.

Given that our ST and snRNAseq analyses consistently indicated that senescence is a central feature of PT biology, we investigated the senolytic activity of dasatinib—a tyrosine kinase inhibitor with established senolytic properties (31). Primary cultures were successfully established from gonadotroph, somatotroph, lactotroph and corticotroph PT. Cells showed high viability before performing dasatinib experiments (**Figure 7A**). We only used β-GAL activity as an indicator of the senescence phenotype, which may be suboptimal as a standalone senescence marker. Approximately 70% of cells from the primary culture metabolized the β-GAL substrate corroborating the presence of a senescence-related state identified by spatial and single nucleus transcriptomics (**Figure 7B**).

Dasatinib was chosen based on our previously reported molecular docking results, in which we described the expression of the DGKG gene in aggressive and recurrent PT, and the electrostatic interaction between DGKG and dasatinib (21). The expression of DGKG and other dasatinib molecular targets was corroborated in two independent cohorts comprising all tumor lineages, widening the actionable range of PT by dasatinib. Here we explore the expression of DGKG and confirmed dasatinib molecular targets such as AKT1, ABL1and PDGFRB (**Supplementary Figure 6**) validating its potential use on these tumors. Exposure to 2.5 μM, and 5 μM dasatinib resulted in a significant, dose-dependent increment in SYTOX staining (reflecting dead cells) and a reduced calcein staining (reflecting viable cells), alongside with morphological changes including alterations in nuclear shape and plasma membrane morphology (**Figure 7C**). Also, we observed activation of caspase 3 and caspase 7 upon dasatinib exposure (**Figure 7D**). When treated with dasatinib, most cells were eliminated including the senescent ones, yielding a reduced cell number and high rates of dead cells and cellular debris.

As previously reported, exposure of GH3 cells to dasatinib resulted in a dose-dependent reduction in cell viability (21). The transcriptomic analysis of these cells revealed that the main molecular events were related to mitochondrial membrane and oxidative phosphorylation (**Supplementary Figure 7**). AtT-20 cells, an ACTH-derived tumor cell line was exposed to dasatinib and caspase 3/7 activation evaluation. Cells treated with dasatinib at 2.5 µM showed a 7.4-fold increase in caspase activity measured by fluorescence, while treatment at 5 µM resulted in a 21.1-fold increase in caspase activity (**Supplementary Figure 8**). This indicates a strong, dose-dependent effect in the number of apoptotic cells. The dasatinib-treated AtT-20 cells exhibited marked morphological alterations, including changes in cell shape and integrity, accompanied by an increase in cellular debris. These findings are consistent with the induction of apoptosis in response to dasatinib treatment.

## 4 Discussion

In the present work we have presented ST as well as snRNAseq evidence showing that aggressive PT consist mostly of terminally differentiated cells that can undergo senescence and growth arrest at phase G1/G0 of the cell cycle. This could partially explain the low proliferative rate of these lesions and could indicate which cells leave the senescence state to start proliferation, thus maintaining tumor mass; senescence could also be implicated in these tumors’ response to pharmacological treatment. Notably, we found no evidence of de-differentiation or EMT.

Cellular senescence is widely used to describe a phenotype characterized by an exit from the cell cycle generally at G1 phase, mainly upon chronic exposure to molecular stress and damage (32, 33). There is evidence that PT in general show a significantly stronger SA-β-GAL staining intensity, compared to normal pituitaries, alongside with other senescence markers (34). Interestingly, POU1F1-derived tumors which include prolactinomas and somatotropinomas, as well as the nonfunctioning tumors of gonadotroph differentiation, show a higher expression of senescence markers (34, 35). Alongside with these results, higher expression of SA-β-GAL was reported in metastatic PT and invasive macroadenomas (36). Estrogen-induced lactotroph PT show the markers of senescence at early stages (37). There is also evidence of PTTG mediated senescence. PTTG regulates p21 expression trough SP1 elements in the p21 promoter, moreover, levels of PTTG expression positively correlate with the levels of aneuploidy in PRL- and GH-producing PT. The consequences of aneuploidy may be proliferation restraint and senescence (7).

Senescence-associated cell cycle arrest limits the propagation of genomic alterations through activation of the DNA damage response, primarily mediated by the p53–p21 axis, thereby preventing the transmission of existing mutations to daughter cells (32). This observation is consistent with our previous longitudinal multi-omics analysis of primary and recurrent PTs, which demonstrated a high degree of genomic stability (21). In addition, senescence can facilitate immune-mediated clearance, collectively contributing to tumor-suppressive effects (32). Cells respond to abnormal oncogene activation with oncogene-induced senescence (OIS), a process that suppresses tumors by halting cell proliferation via regulators like p53 and p16INK4a (38). Overexpressed oncogenes trigger replicative stress, activating pathways that lead to growth arrest. Senescence can also result from loss of tumor suppressor genes; for example, PTEN loss causes senescence by activating AKT and mTOR, which then increase p53 and p16INK4a levels to limit tumor growth (38).

Therapy-induced senescence (TIS) is a type of premature senescence resulting from the exposure to chemotherapeutic agents or radiation which cause genotoxic stress (39). Activation of the p53-dependent G1 cell cycle checkpoint is essential in TIS, however p53-negative cancer cells can also be driven into senescence (40). TIS may exert beneficial effects by acting as a barrier to tumor progression and by promoting activation of the innate immune system, thereby facilitating the clearance of malignant clones (40). Conversely, TIS can also have deleterious consequences, by reshaping the tumor microenvironment and overcoming growth-restraining immunosuppressive signals, it may enhance cellular migration and invasion, contributing to the establishment of a pre-metastatic niche (40). Moreover, TIS has been implicated in therapy resistance through the promotion of pro-survival pathways, EMT, and stemness programs (40). However, in our study, we did not identify transcriptomic evidence supporting the activation of EMT or stemness-associated signatures.

The PI3K-AKT pathway has been implicated in senescence in several cell types such as endothelial cells, embryonic fibroblast and keratinocytes mainly through regulation of mTORC1, p53, p21 and p19ARF among others (41). Chronic PI3K/AKT/mTORC1 pathway activation promotes a specific type of OIS known as AKT-induced senescence (AIS). In this context, senescence is triggered by PIK3CA mutants, PTEN knockdown, and constitutively active AKT (42). Unlike OIS, AIS lacks a hyperproliferative burst and DNA damage, and proliferative arrest relies on p53 rather than p16 (42). One interesting fact is the absence of SASP-related markers, which could indicate that OIS, TIS or AIS could be having an effect, nevertheless, more research is needed to further elucidate this absence.

We have previously demonstrated that transcriptomically PT are characterized by alterations in lipid synthesis and metabolism (15, 21). The most prominently altered lipids during the senescent process are phosphatidyl inositol (PI) and phosphatidyl glycerol (PG) although mitochondrial lipids are also affected (43). The changes in lipid metabolism in senescent cells are mainly concentrated in the metabolic processes of phospholipids, fatty acids and cholesterol (44). The changes in lipid-metabolizing enzymes and proteins involved in these pathways play a critical role in senescence (44).

Although we did find evidence supporting that the senescence process is activated in PT as indicated by the expression of β-GAL, we cannot rule out that the observed changes may reflect quiescence or terminal differentiation. Senescence is an irreversible cell cycle arrest induced by DNA damage or aging cells, whereas quiescence is a reversible cell cycle arrest that occurs due to nutrition and/or growth factors depletion (45). Senescence and quiescence are influenced by p53 activation and duration of such activation (45). While maximal activation of p53 inhibits mTOR and leads to quiescence, partial p53 activation results in mTOR preservation and subsequently in senescence which in turn promotes p21 and p27 among other cyclin dependent kinase inhibitors (45). The cell cycle arrest that characterizes quiescence, which can be promoted by CDK inhibitors, constitutes only one part of the senescence process (45). Terminal differentiation is usually associated with a permanent exit from the cell cycle and represents the most common cellular state (46). Cell cycle exit invoke induces repression of Cyclin/Cdk activity by cyclin dependent kinase inhibitors (CKI) such as p21, p27 and p57, or repression of E2F-mediated transcription by members of the retinoblastoma (Rb) family. Senescence, quiescence and terminal differentiation are all characterized by a prolonged cell cycle arrest at G1 phase of the cell cycle, as well as by the presence of hypophosphorylated Rb proteins which inhibits E2F activity and often results in high CKI activation (46). Senescence, quiescence and terminal differentiation share several molecular pathways which hampers the discrimination between these processes.

Notably, senescence represents a potential therapeutic target, as the associated alterations in cellular physiology can be used for the selective elimination of senescent cells (senolysis) (31). Senolytics comprise a class of agents designed to preferentially induce apoptosis in senescent cells. Early senolytic strategies included combination therapies such as dasatinib and quercetin, which target multiple pro-survival pathways, including ephrin signaling, PI3K, and AKT, among others (47, 48). In the present manuscript and our previous work and in our previous investigations (15, 21) we demonstrated the expression of several dasatinib targets in PT including PI3K/AKT pathway related genes, which are also altered in the present samples. Also, we have previously reported molecular docking results of dasatinib electrostatic interaction with targetable genes in PT (21). It remains unclear whether senescence arises prior to, during, or following pharmacological treatment and/or surgical intervention, or if it is intrinsically acquired during tumor development. We are also currently analyzing other mechanisms that could be responsible for the relatively slow proliferation rate of PT.

There is evidence that imatinib and dasatinib may disrupt the GH-IGF1 (insulin-like growth factor 1) axis (49). This effect has not only been observed in murine models, since pediatric patients with leukemia treated with this TKI frequently showed growth retardation associated with a down regulation of the somatotropic axis (50, 51). If this holds true, dasatinib could not only eradicate pituitary tumoral cells, but it could also inhibit growth hormone secretion making it an attractive therapeutic alternative in hormone secreting pituitary lesions.

High-throughput data from genomic, transcriptomic, proteomic, and metabolomic studies had significantly contributed important knowledge not only in to the molecular pathogenesis of PT, but also had allowed the emergence of drug repurposing and mechanics-based drug discovery into the context of personalized and precision medicine (52). Bioinformatic analysis can expedite the identification of drug targets, enhance the screening and optimization of drug candidates, and facilitate the characterization of side effects and prediction of drug resistance (52). An example of such approach is the use of this information in the discovery and design of new drugs for the treatment of PT, using data derived from RNA sequencing and high-throughput drug screening have help identified the NRF2 gene as a therapeutic target of the histone deacetylase agent panobinostat (53).

In conclusion, PT consist of terminally differentiated cells with a senescent phenotype at different proportions, that can be targeted with dasatinib, a TKI with senolytic properties. Yet, we must acknowledge the limitations of our study which include a relatively small sample size and perhaps an unavoidable underrepresentation of GH-, PRL- and ACTH-secreting tumors. Also, we must acknowledge the different technologies used in this protocol, and that such differences could be reflected in the precision of the methods used to estimate the senescence score, since the ST version used has no single cell resolution whereas snRNAseq does have higher resolution, further research is needed, for which we are currently working on the spatial characterization of other PT lineages. Also, we acknowledge the lack of understanding of the mechanism of how dasatinib work in pituitary tissue, for which we are currently exploring the mechanism of action of dasatinib as a senolytic drug and if and how it can regulate hormone expression and secretion. Currently we cannot discern between senescence, quiescence and terminal differentiation cell cycle arrest, and thus further basic and translational research is needed to address and understand the molecular events underlaying senescence.

## Data Availability

Due to governance restrictions, the datasets generated and analyzed during the current study are deposited in an institutional repository with accession number A290224 and access is available from the corresponding author upon reasonable request. This due to the sensitive nature of the clinical data, genomic and transcriptomic information that could lead to patient identification and was requested by our local ethical committee. Data from the gonadotroph snRNAseq was deposited have been deposited at GEO with accession number GSE295840.
