# Supplementary figures and images for: Analysis of senescence in pituitary tumors from different lineages and the potential role of senolytic drugs as targeted therapies
Source: Front Endocrinol (Lausanne). 2026 Jul 15;17:1836525. doi: 10.3389/fendo.2026.1836525 (PMC13414927; doi:10.3389/fendo.2026.1836525)

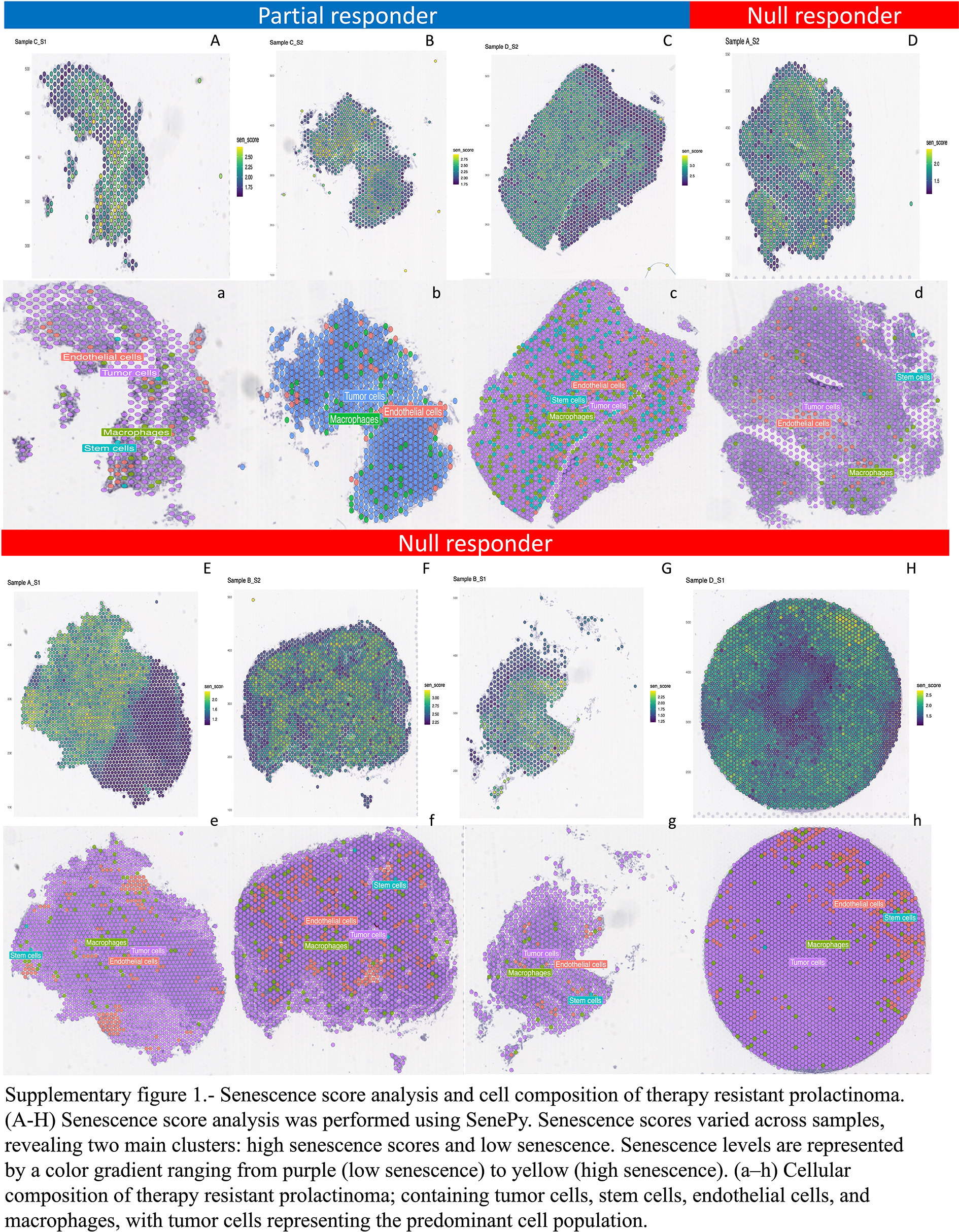

Supplement: Supplementary file 1 [file Image1.jpeg]

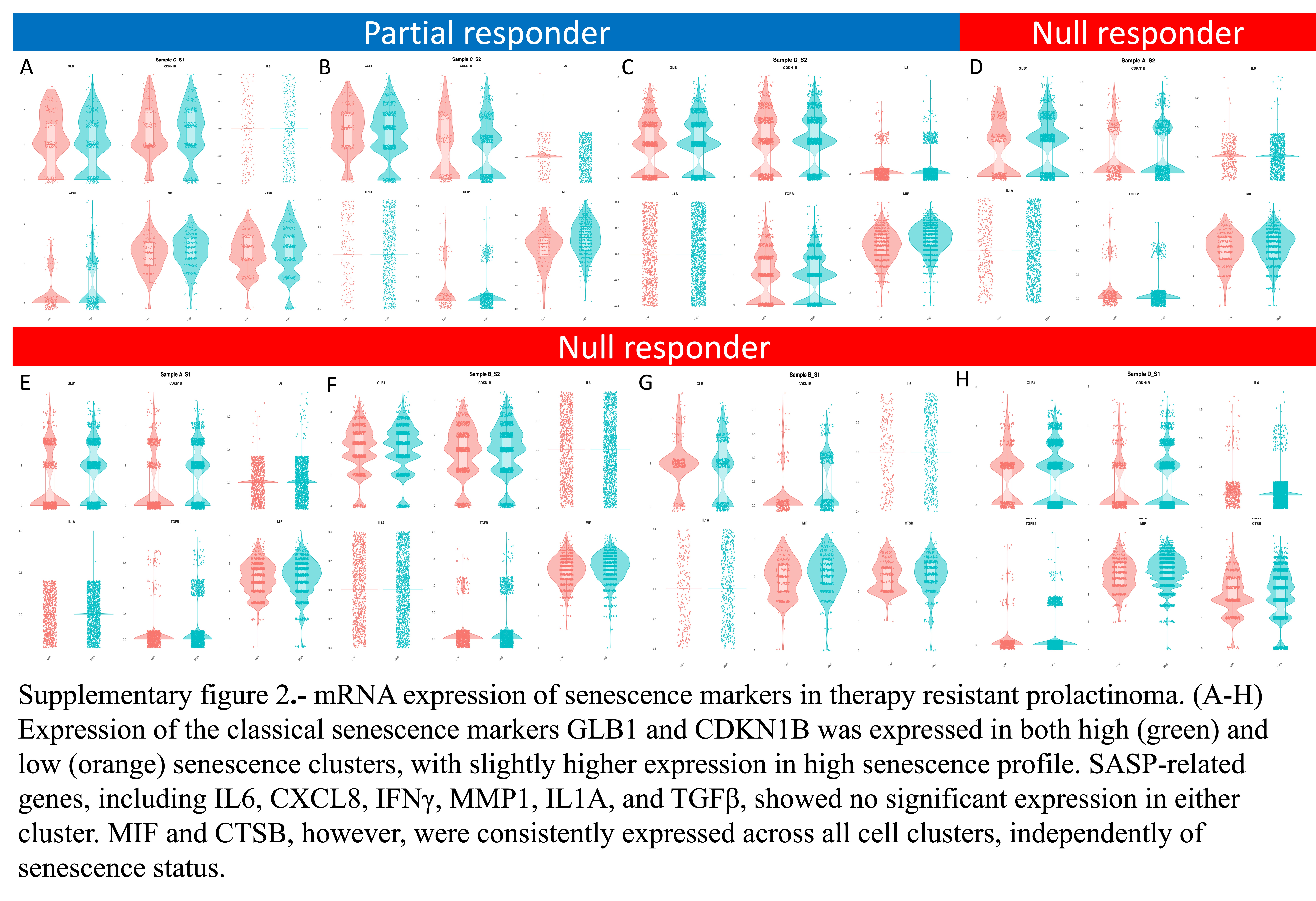

Supplement: Supplementary file 2 [file Image2.jpeg]

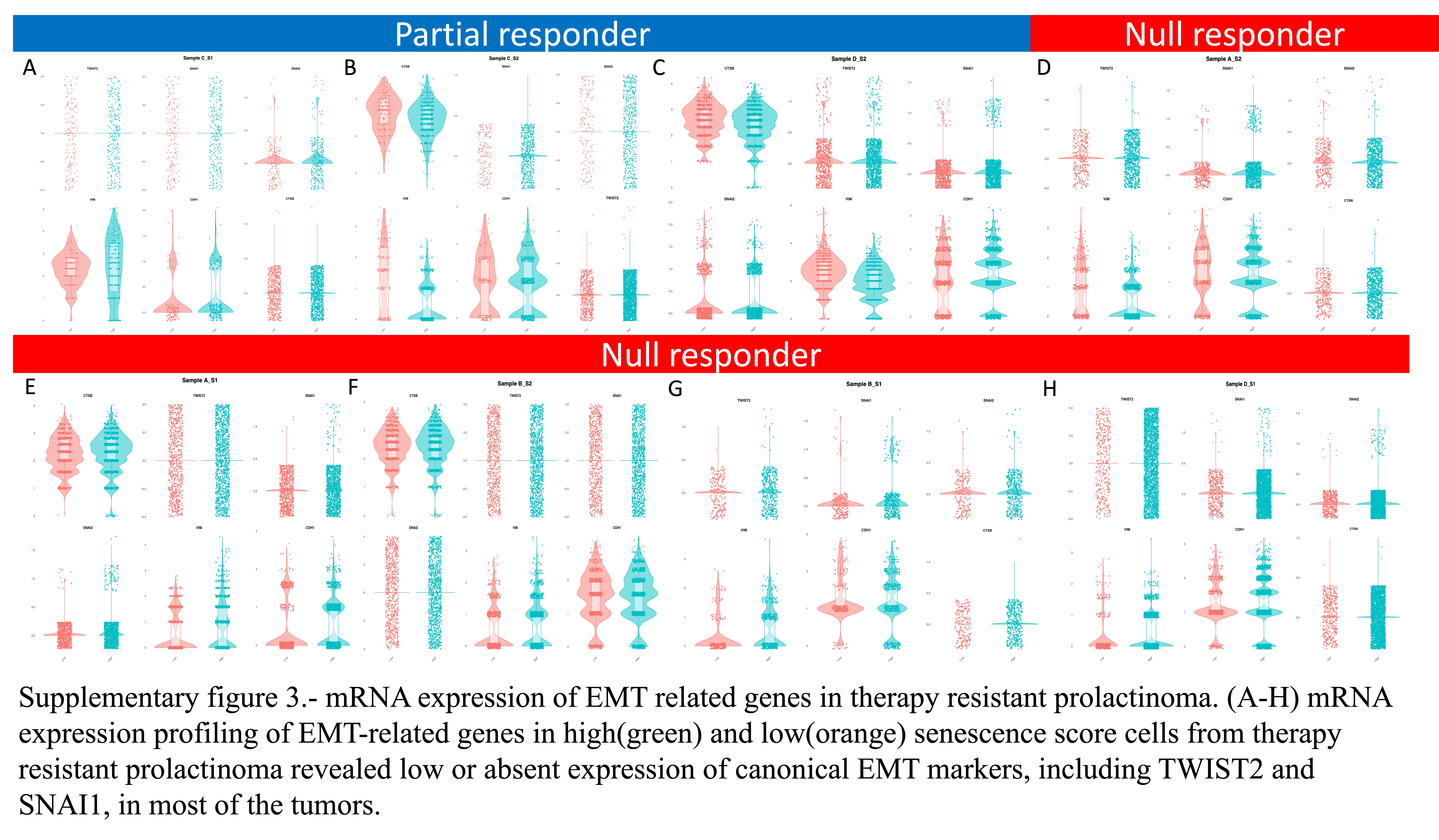

Supplement: Supplementary file 3 [file Image3.jpeg]

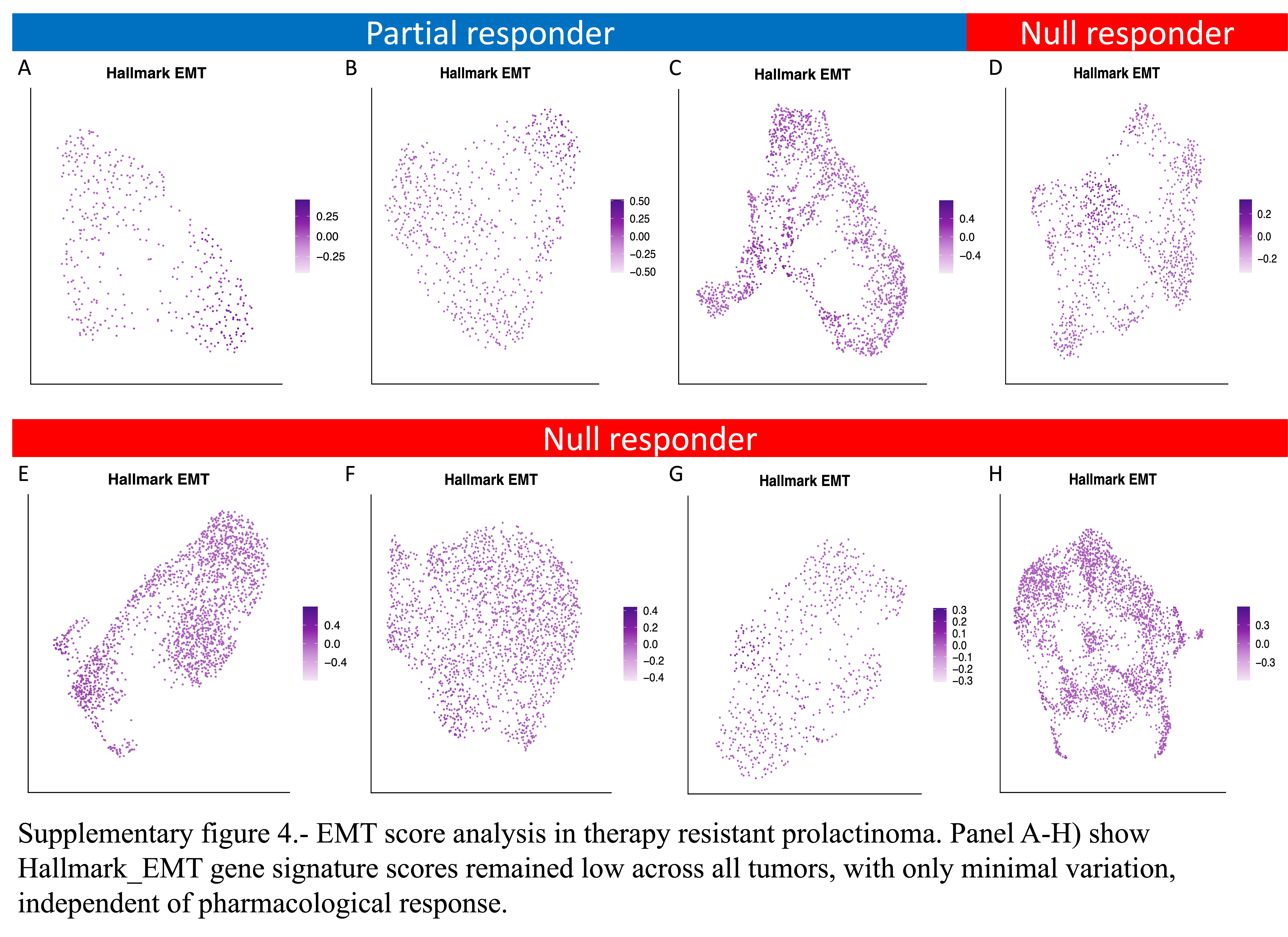

Supplement: Supplementary file 4 [file Image4.jpeg]

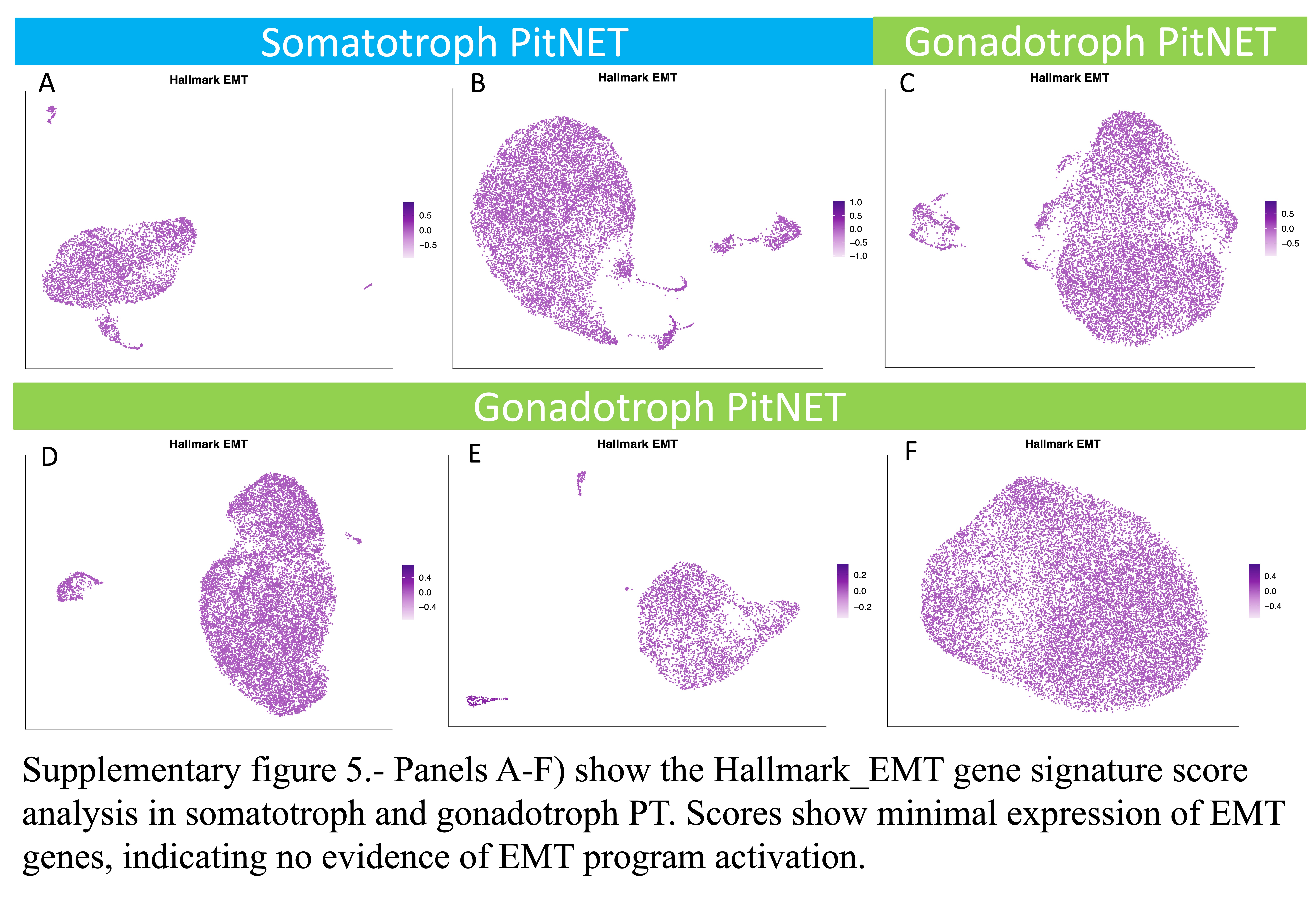

Supplement: Supplementary file 5 [file Image5.jpeg]

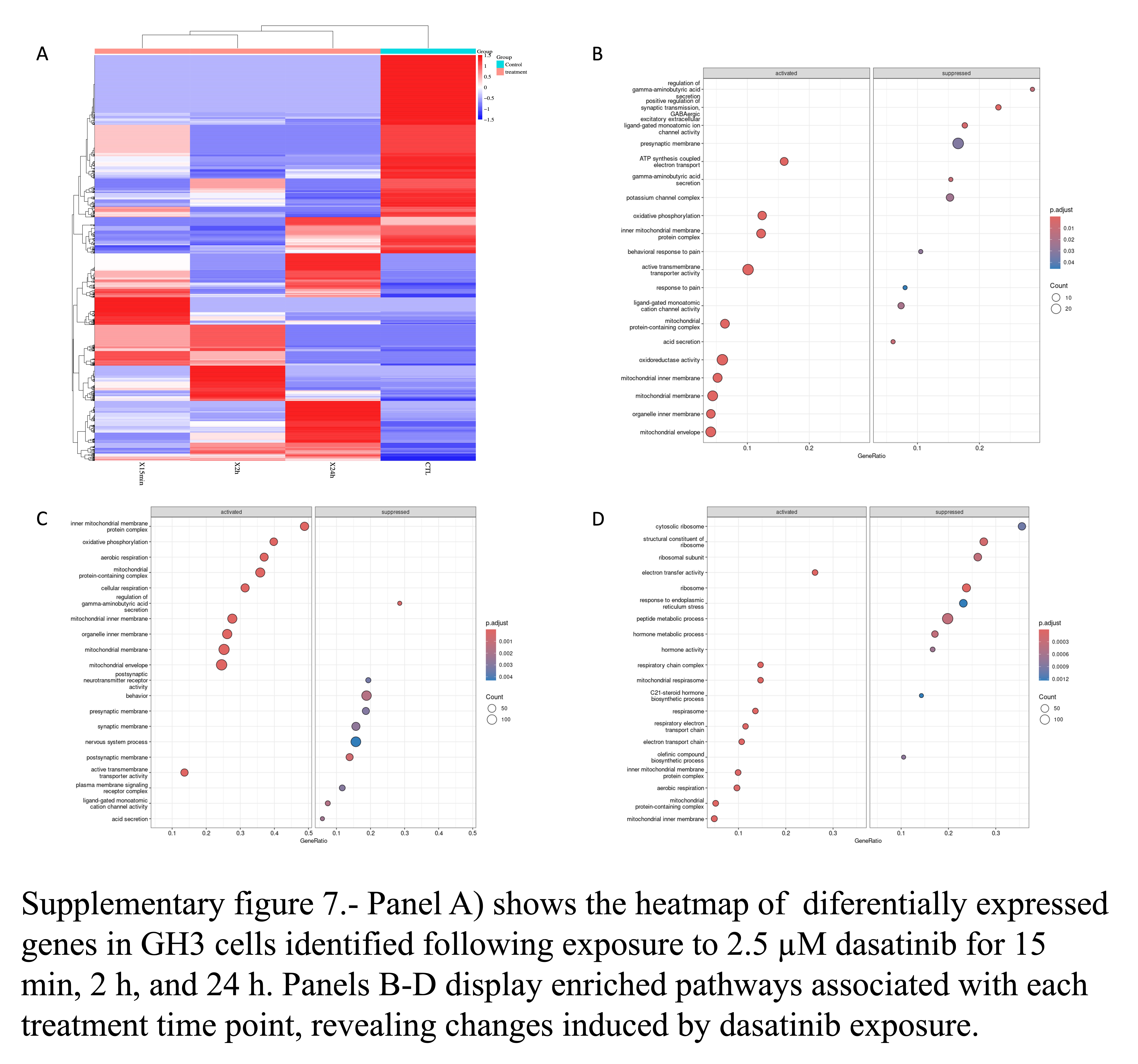

Supplement: Supplementary file 7 [file Image7.jpeg]

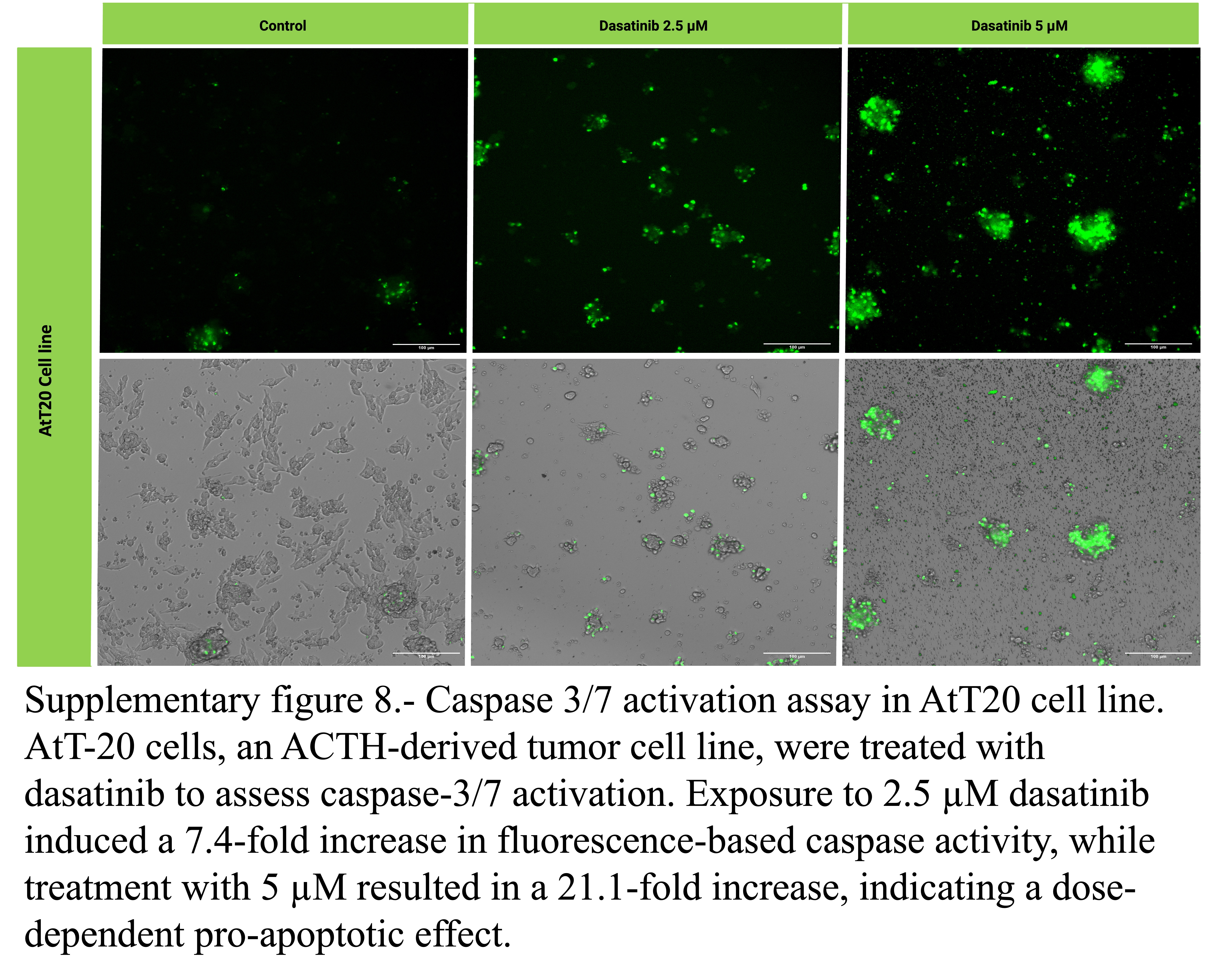

Supplement: Supplementary file 8 [file Image8.jpeg]
